# Epidemiologic Trends in Malaria Incidence Among Travelers Returning to Metropolitan France, 1996-2016

**DOI:** 10.1001/jamanetworkopen.2019.1691

**Published:** 2019-04-05

**Authors:** Eric Kendjo, Sandrine Houzé, Oussama Mouri, Aida Taieb, Frédérick Gay, Stéphane Jauréguiberry, Ilhame Tantaoui, Papa A. Ndour, Pierre Buffet, Martine Piarroux, Marc Thellier, Renaud Piarroux

**Affiliations:** 1 Sorbonne Université, Institut National de la Santé et de la Recherche Médicale (INSERM), Institut Pierre–Louis d’Epidémiologie et de Santé Publique, Assistance Publique Hôpitaux de Paris (AP-HP), Centre National de Référence du Paludisme, Hôpital Pitié–Salpêtrière, France; 2 Sorbonne Université, Institut de Recherche pour le Développement, AP-HP, Centre National de Référence du Paludisme, Hôpital Bichât Claude–Bernard, Paris, France; 3 AP-HP, Centre National de Référence du Paludisme, Hôpital Pitié–Salpêtrière, Paris, France; 4 Sorbonne Université, INSERM, Laboratory of Excellence GR–Ex The Red Blood Cell, Paris, France; 5 Sorbonne Université, INSERM, Laboratory of Excellence GR–Ex The Red Blood Cell, Institut National de la Transfusion Sanguine, AP-HP, Hôpital Necker–Enfants Malades, Paris, France; 6 Sorbonne Université, INSERM, Institut Pierre–Louis d’Epidémiologie et de Santé Publique, AP-HP, Hôpital Saint–Antoine, Paris, France

## Abstract

**Importance:**

Despite annually adapted recommendations to prevent malaria in travelers to endemic areas, France is still the industrialized country reporting the highest number of imported cases of malaria. Better understanding of the epidemiologic context and evolution during the past 2 decades may help to define a better preventive strategy.

**Objective:**

To study epidemiologic trends of imported cases of malaria in travelers in geographic territories of France on the European continent (metropolitan France) from 1996 through 2016 to potentially explain the persistence of high imported malaria incidence despite national preventive measures.

**Design, Setting, and Participants:**

In a cross-sectional study, between January 1 and May 31, 2018, data were extracted from the French National Reference Center of Malaria Surveillance. Trends in patients with imported malaria in association with age, sex, ethnicity, purpose of travel, malaria species, severity of illness, case mortality rate, and endemic countries visited were analyzed in 43 333 malaria cases among civilian travelers living in metropolitan France.

**Main Outcomes and Measures:**

Evolution of the main epidemiologic characteristics of patients with imported malaria.

**Results:**

Among the 43 333 patients with imported malaria in civilian travelers included in the study, 24 949 were male (62.4%), and 8549 were younger than 18 years (19.9%). A total of 28 658 malaria cases (71.5%) were among African individuals, and 10 618 cases (26.5%) among European individuals. From 1996 through 2016, the number of confirmed malaria cases peaked at 3400 cases in 2000, then declined to 1824 cases in 2005 and stabilized thereafter to approximately 1720 malaria cases per year. A total of 37 065 cases (85.5%) were due to *Plasmodium falciparum*. The proportion of malaria cases among African individuals rose from 53.5% in 1996 to 83.4% in 2016, and the most frequent motivation for traveling was visiting friends and relatives (25 329 [77.1%]; *P* < .001). Despite an increase in the proportion of severe cases, which rose from 131 cases (8.9%) in 1996 to 279 cases (16.7%) in 2016 (*P* < .001), mortality remained stable, being approximately 0.4% during the study period.

**Conclusions and Relevance:**

Beyond the apparent stability of the number of imported malaria cases in France, significant changes appear to have occurred among the population who developed malaria infection following travel in endemic areas. These changes may imply that adaptation of the preventive strategy is needed to reduce the burden of the disease among travelers.

## Key Points

**Question:**

Could changes in the population experiencing imported malaria in France over the past 2 decades explain the persistence of the high number of malaria cases despite national preventive measures?

**Findings:**

In this cross-sectional study of 43 333 malaria cases from travelers returning to France from a malaria-endemic area, the proportion of malaria cases among African individuals has increased significantly from 1996 through 2016 (53.5% vs 83.4%).

**Meaning:**

Although prophylactic measures appeared to be efficient among European individuals traveling for tourism or business purposes, progress is needed to ensure better protection for African individuals visiting friends or relatives.

## Teaser

This cross-sectional study of data from the French National Reference Center of Malaria Surveillance examines the epidemiologic characteristics associated with imported cases of malaria among travelers returning to metropolitan France.

## Introduction

France remains the European country reporting the highest number of imported malaria cases,^1^ with approximately 2000 cases being reported annually by the Centre National de Référence du Paludisme (CNR du Paludisme),^2^ which is the National Reference Center of Malaria Surveillance.^3^ Moreover, 4 national surveys have shown that cases reported by the CNR du Paludisme surveillance system account for 50% to 55% of all French malaria cases, leading to an estimated number of cases exceeding 4000 each year.^3,4^

This persistence of a high number of imported malaria cases in France contrasts with the amount of preventive means to reduce the risk of malaria among travelers.^4,5^ These means include the dissemination of prevention messages, personal antivector protection, and chemoprophylaxis adapted to the context of the stay, which may be costly for travelers in the event of prolonged stay in malaria-endemic areas.^6,7,8,9^ Similarly, the purchase of protective equipment against mosquito bites remains the responsibility of travelers, increasing the cost of travel.^10,11^ These additional costs may be an obstacle for travelers, especially those returning to their country of origin as visitors of friends and family (VFR).^9,12,13^ The standard of living of immigrant households has been shown to be lower than that of nonimmigrant households in France.^14,15^ We hypothesize that a change in the epidemiologic characteristics of travelers, and particularly an increasing proportion of VFRs,^16^ may be associated with the persistence of a high malaria incidence in France. This study was therefore conducted to describe trends in epidemiologic characteristics of imported malaria in geographic territories of France on the European continent (metropolitan France)^17^ from 1996 to 2016.

## Methods

### Participants

Only patients with malaria diagnosed in metropolitan France from civilian travelers were included to avoid biases related to military, people living in endemic areas, or autochthonous malaria with a specific epidemiology. This study followed the Strengthening the Reporting of Observational Studies in Epidemiology (STROBE) reporting guideline.^18^ Data collection and storage by the French National Reference Center of Malaria Surveillance System database (CNR du Paludisme) were approved by the French National Commission for Data Protection and Liberties. The study was approved by the Ethics Committee for Biomedical Research with a waiver of specific consent by the patients. The collected data were deidentified and analyzed according to the French National Public Health Agency guidelines.^19^

### Study Design and Data Sources

We conducted a retrospective analysis of serial case cross-sectional data available from the CNR du Paludisme. We analyzed trends in imported malaria cases in association with age, sex, ethnicity (African vs European individuals), purpose of travel, species of malaria, severity of illness (severe or uncomplicated malaria), case mortality rate, and endemic countries visited. The metropolitan France CNR du Paludisme is organized into 3 reference laboratories—1 in Marseille and 2 in Paris—along with a network of approximately 100 hospitals throughout the national territory, which fully report cases to a secured database and send blood samples to reference laboratories.

### Case Definition

All suspected cases were confirmed by microscopy or polymerase chain reaction either at the network hospitals or by the CNR du Paludisme reference laboratories. Case definition of malaria and reporting methods did not change over the study period. However, a severe malaria case was first defined according to World Health Organization criteria for severe malaria before 1999, then modified by the 1999 French National Consensus on Malaria, and revised in 2007.^4,5,20^ Thus, we have homogenized the definition of severe cases according to the 2007 revision of the 1999 Consensus Conference for Malaria for all of the study period.

### Diagnosis and Case Managements

Malaria diagnosis was confirmed by physician and biologist experts involved in everyday diagnostic procedures, including species identification, parasitemia count, and treatment monitoring. Information regarding the cases was provided using a secured online database^21^: social and demographic information (date of birth, sex, country of birth, country of residence, ethnicity), details of travel (date of arrival in metropolitan France, country visited, purpose of travel, length of stay, prophylaxis used), and details of illness (date of onset of symptoms, method of diagnosis, date of treatment, first- and second-line treatment).^19^

The variable ethnicity had 3 categories: African individuals if at least 1 parent was African born, European individuals if both parents were born in Europe, and other individuals. Owing to the heterogeneity of the other individuals category, we concentrated our analysis on both African and European people. Within the African individuals category, a subgroup called African VFRs was assessed. African VFRs were considered African individuals living in France who return to their home country to visit friends and family.^16^

### Estimation of the Total Number of Malaria Cases

The total incidence of *Plasmodium* malaria infection was derived using 4 complete national surveys performed in 1997, 1999, 2004, and 2013 (eMethods in the Supplement).^3^

### Statistical Analysis

Between January 1 and May 31, 2018, we extracted and analyzed data samples available from the French National Reference Center of Malaria Surveillance System. We used descriptive statistics to summarize the data. Quantitative variables are represented by medians (interquartile range [IQR]). Categorical variables are characterized as frequencies. Fisher exact tests or Pearson χ^2^ analysis were used to assess differences in demographic and clinical characteristics. The Wilcoxon test was used to compare 2 independent groups not normally distributed, and the Kruskal-Wallis test was used to test more than 2 groups. The box and whisker plot was used to visualize data distribution through quartiles. The Kernel density estimation was used to create smooth curves to assess temporal changes in the number of malaria cases over time. Simple linear regression was applied to test for linear temporal trend. Sensitivity analyses were performed to compare variables with or without missing data. For each variable, missing data were defined as the absence of case record and unknown data if the case recorded mentioned that, in that case, the variable was not tested for. A difference was considered significant at *P* < .05, except for multiple comparisons between groups when Bonferroni correction was performed to avoid α risk inflation. All reported *P* values were 2-tailed. Statistical analyses were performed using JMP pro, version 13.1 (SAS Institute Inc).

## Results

### General Description

**Figure 1.**
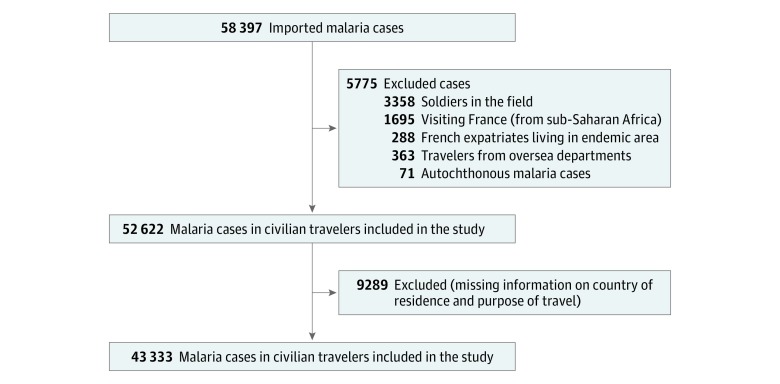
Flowchart of Imported Malaria in Civilian Travelers, Metropolitan France, 1996-2016 Malaria cases were identified through the Centre National de Référence du Paludisme.

A total of 58 397 malaria cases were reported to the CNR du Paludisme from 1996 through 2016; 5775 of those outside our study scope were excluded (Figure 1). We also excluded 9289 cases with missing information on residence and purpose of travel. Sensitivity analyses showed that the order of magnitude of differences between cases with or without missing values ranged from 1 to 5 percentage points, except for ethnicity. Of these cases, 43 333 fit the inclusion criteria (Figure 1), including 8549 children younger than 18 years (19.9%) and 24 949 (62.4%) male.

### Imported Malaria Trend

**Figure 2.**
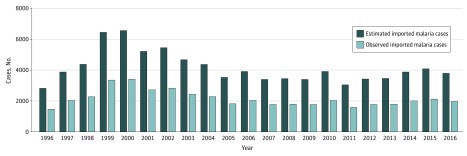
Imported Malaria Cases In Civilian Travelers, Metropolitan France, 1996-2016 Observed and estimated cases.

As reported in Figure 2, from 1996 through 2016, the number of confirmed malaria cases peaked at 3400 in 2000 and then declined to 1824 in 2005. The number stabilized thereafter to approximately 1720 malaria cases per year.

### Species Diagnosis

Among the 43 333 cases with species reported, *Plasmodium falciparum* was the most frequently encountered, being 37 065 cases (85.5%), followed by *Plasmodium ovale* (2405 [5.6%]), *Plasmodium vivax* (1732 [4.0%]), *Plasmodium malariae* (748 [1.7%]), and mixed infections (736 [1.7%]). Simple linear regression showed a significant increase in the number of *P falciparum* infections, ranging from 1200 cases (81.9%) in 1996 to 1448 cases (86.8%) in 2016 (*P* < .001).

Among 43 285 imported cases with species and regions of transmission documented, 41 780 cases (96.5%) were acquired in sub-Saharan Africa, with a significant increase in the number of cases ranging from 1178 cases (84.8%) to 1422 cases (88.3%), respectively, in 1996 and 2016 (*P* < .001). The *P falciparum* species accounted for 36 603 (87.6%) of the cases acquired in sub-Saharan Africa, 206 (29.7%) of the cases acquired in Latin America and the Caribbean, and 157 (22.7%) of the cases acquired in Asia. Most of the *P vivax* cases reported were acquired in Asia (938 of 1726 [54.3%]), 431 (25.0%) were acquired in Latin America and the Caribbean, and 321 (18.6%) were acquired in North and East Africa. Almost all *P ovale* (2368 [98.6%]) and *P malariae* (731 [98.2%]) infections were acquired in sub-Saharan Africa.

### Seasonality

The monthly distribution of malaria cases was species dependent with a dominant peak between August and October of 17 798 cases of *P falciparum* (48.1%). A similar but less pronounced pattern was observed for *P malariae*. The incidence of *P vivax* and *P ovale* was relatively stable over the years (eFigure 1 in the Supplement).

### Visited Countries

The 10 most reported countries for malaria transmission were in sub-Saharan Africa and represented 34 726 of the 43 285 total cases (80.2%). Most of the countries were in Western Africa (Ivory Coast, Mali, Senegal, Burkina-Faso, and Guinea) (21 093 [48.7%] cases), followed by Central Africa (Cameroon, Republic of the Congo, and Central African Republic) (9105 [21.0%]) and the Indian Ocean (Republic of Comoros) (3929 [9.1%]) (eFigure 2 in the Supplement).

### Age Distribution and Sex Ratio

**Figure 3.**
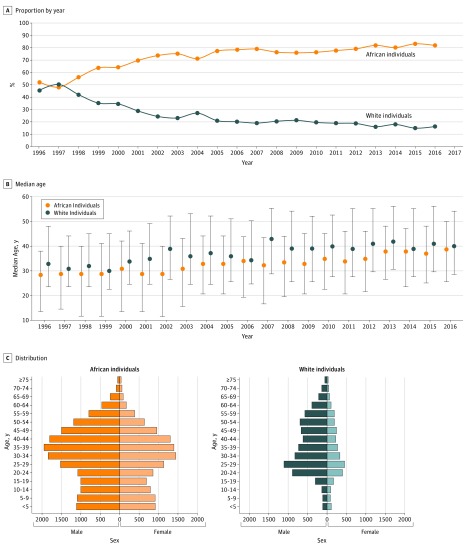
Civilian Travelers With Imported Malaria in Metropolitan France, 1996-2016 A, Evolution of the proportion of African vs European travelers by year (n = 39 276). B, Median age (n = 42 971); error bars indicate interquartile range. C, Distribution of age by sex (n = 42 905).

The median age of the patients (n = 42 971) was 33 (interquartile range [IQR], 21-45) years, ranging from 0 to 96 years. Simple linear regression showed that there was a significant aging of individuals diagnosed with imported malaria during the study period, with a median increase 8 years from 1996 through 2016 (*P* < .001). A shift in age distribution between 1996 and 2016 was observed with an increase in the number of cases occurring in the 45 to 60 years and older than 60 years classes (eFigure 3 in the Supplement). Six hundred fifty infants (0-1 year) represented 1.5% of all imported cases; children (2-14 years: 6071 [14.1%]), adults (15-59 years: 33 879 [78.8%]), and those older than 60 years (2371 [5.5%]) accounted for the remainder of the cases (Figure 3C).

Overall, the sex ratio (male to female) was 1.7 during the study period. The ratio significantly increased with age, ranging from 1.0 in infants to 2.8 in patients older than 60 years (*P* < .001).

### Ethnicity

Overall, the number of malaria cases among African and European individuals was 39 276. Most patients were African (28 658 [71.5%]) or European (10 618 [26.5%]) (Table). Between 1996 and 2016, there was a significant increase in the proportion of malaria cases in African individuals (1996: 665 [53.5%]) and 2016: 1242 [83.4%]), with a mirrored decrease in European individuals (1996: 578 [46.5%] and 2016: 247 [16.6%]) (Figure 3A). The distribution of age by sex was known for 42 905 individuals. European individuals were significantly older than African individuals, with median ages of 35 (IQR, 25-50) and 33 (IQR, 18-44) years (*P* < .001) (Figure 3B). During the study period, there were significant trends in aging for both African and European individuals. The median age was 29 (IQR, 14-38) years in 1996 and 39 (IQR, 26-50) years in 2016 for African individuals (*P* < .001), and 33 (IQR, 24-48) years in 1996 and 40 (IQR, 29-54) years in 2016 for European individuals (*P* < .001). The sex ratio was significantly higher in European individuals than African individuals (2.5 vs 1.4; *P* < .001) (Figure 3C).

**Table.** Epidemiologic Characteristics of Imported Malaria Cases in Civilian Travelers by Ethnicity, Metropolitan France, 1996-2016

Variables	No. (%)^a^				*P* Value
	All (N = 43 333)^b^	African Individuals (n = 28 658)	European Individuals (n = 10 618)	Other Individuals (n = 783)	
Demographic characteristic					
Age, median (IQR), y	33 (21-45)	33 (18-44)	35 (25-50)	31 (20-43)	<.001^c^
Sex ratio, male to female	1.7	1.4	2.5	2.2	<.001^d^
Purpose of travel					
Visiting friends and relatives	25 329 (77.1)	24 928 (93.7)	168 (2.9)	233 (44.5)	<.001
Tourism	3757 (11.4)	563 (2.1)	3064 (53.7)	130 (24.8)	
Business	2356 (7.2)	430 (1.6)	1836 (32.2)	90 (17.2)	
Humanitarian assistance	182 (0.6)	26 (0.1)	152 (2.7)	4 (0.8)	
Air or Merchant Navy crew	129 (0.4)	21 (0.1)	100 (1.8)	8 (1.5)	
Backpacking trip	106 (0.3)	11 (0.0)	89 (1.6)	6 (1.1)	
Other	980 (3.0)	627 (2.4)	300 (5.3)	53 (10.1)	
Diagnosis					
* Plasmodium falciparum*	34 300 (85.6)	25 497 (89.0)	8287 (78.0)	516 (65.9)	<.001
* Plasmodium vivax*	1591 (4.0)	402 (1.4)	984 (9.3)	205 (26.2)	
* Plasmodium ovale*	2226 (5.6)	1432 (5.0)	776 (7.3)	18 (2.3)	
* Plasmodium malariae*	674. (1.7)	482 (1.7)	178 (1.7)	13 (1.7)	
Mixed infection	674 (1.7)	452 (1.6)	199 (1.9)	23 (2.9)	
*Plasmodium* spp	594 (1.5)	393 (1.4)	193 (1.8)	8 (1.0)	
Prevention					
Alleged chemoprophylaxis	16 430 (44.1)	10 833 (40.9)	5428 (53.7)	169 (24.5)	<.001^d^
Clinical outcome					
Severe malaria	2462 (6.2)	1350 (4.8)	1097 (10.1)	83 (10.7)	<.001
Uncomplicated malaria	37 110 (93.8)	26 931 (95.2)	9489 (90.2)	690 (89.3)	
Died	153 (0.4)	40 (0.1)	106 (1.0)	7 (0.9)	<.001

Abbreviation: IQR, interquartile range.

^a^ For each variable, missing data were defined as the absence of case record and unknown data if the case record mentioned that, in that case, the variable was not tested for. Sensitivity test comparing the 9289 cases with missing information on country of residence and purpose of travel vs the remaining 43 333 malaria cases was done. This analysis showed stability of our results, with significant differences not clinically relevant. The order of magnitude ranged from 1 to 5 percentage points.

^b^ Data on ethnicity were available in 40 059 (92.4%) of the 43 333 cases of the study.

^c^ Wilcoxon test.

^d^ Pearson χ^2^ test, Fisher exact test.

**Figure 4.**
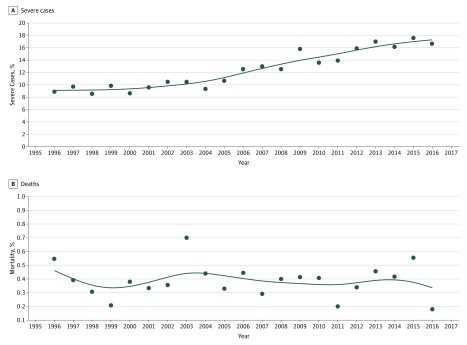
Severity and Mortality in Civilian Travelers With Imported Malaria in Metropolitan France, 1996-2016 A, Severe imported malaria cases (n = 5158). B, Case-fatality rates (n = 166).

Concerning the purpose of travel, full information, including that on tourism and business, was not available before 2000. Overall, the most frequent motivation for traveling was VFRs (25 329 [77.1%]; *P* < .001), which increased to 79.6% in 2016. African individuals were primarily VFRs (24 928 [93.7%]). In contrast, European individuals were mostly tourists (3064 [53.7%]) and business travelers (1836 [32.2%]) (Table). Moreover, 3332 of 4199 of imported malaria cases (79.4%) reported in France from 2014 through 2016 were attributed to African VFRs compared with 777 of 4199 imported malaria cases (18.5%) for European tourists (Figure 4A). European individuals are often male and either business travelers or tourists (4693 [82.2%]); African individuals more often travel as a family, with children of all age groups (sex ratio close to 1) and significantly longer lengths of stay compared with European individuals (6 [IQR, 4-8] vs 4 [IQR, 2-8] weeks; *P* < .001) (Figure 3C, eTable 1 in the Supplement).

### Delay Before Diagnosis

Time between return and onset of symptoms and time between onset of symptoms and diagnosis were associated with species. The median time between return and onset of symptoms was 86 (IQR, 17-201) days for *P ovale*, 65 (IQR, 11-177) days for *P vivax*, 24 (IQR, 5-54) days for *P malariae*, 7 (IQR, 1-26) days for mixed infections, and 5 (IQR, 0-11) days for *P falciparum*. The median time between onset of symptoms and diagnosis was 7 (IQR, 3-16) days for *P malariae*, 4 (IQR, 1-7) days for *P vivax*, 4 (IQR, 2-7) days for *P ovale,* 4 (IQR, 2-7) days for mixed infections, and 3 (IQR, 2-6) days for *P falciparum*. For *P falciparum*, 31 990 cases (98.2%) were diagnosed within 2 months of return. The time between onset of symptoms and diagnosis did not change significantly for *P falciparum* over time (eFigure 4 in the Supplement)

### Severe Cases and Mortality

Overall, severe cases (n = 5158) increased from 131 of the imported malaria cases in 1996 (8.9%) to 279 cases in 2016 (16.7%) (*P* < .001) (Figure 4A). This increase was not associated with an elevation in mortality, which remained stable at approximately 0.4% (n = 166) during the study period (Figure 4B).

## Discussion

Our findings show changes in the population structure of people with imported malaria in metropolitan France, which may explain the persistence of a higher level of malaria incidence, particularly of severe cases. The most striking changes were the increase in the proportion of malaria cases among African individuals (from 53.5% in 1996 to 83.4% in 2016, most of them African VFRs, to 79.6% in 2016) (Figure 4A), the 8-year increase of the median age of the patients (African, 10 years and European, 7 years) and the doubling in the proportion of severe cases. These changes were supported by the fact that modalities of our surveillance system remained unchanged and the network of participating hospitals was stable over the study period.

Our study shows that malaria cases peaked at 3400 in 2000 and then declined until to 2005, followed by stability at approximately 1720 cases per year until 2016 (Figure 2). A global decrease in the imported malaria incidence was observed in many industrialized countries, such as Denmark, Switzerland, the Netherlands, Italy, Germany, and the United Kingdom.^22,23,24,25,26,27^ The reduction in the parasite transmission in malaria-endemic areas^28^ and chemoprophylaxis effectiveness could explain this trend, because several authors reported that the better efficacy of the atovaquone with proguanil (available in France since 1998) combination in prophylactic use is due to better adherence to the administration regimen schedule.^29,30,31^ Other studies, however, reported an increase of imported malaria incidence, gradually in Spain or the United States and more sharply in recent years in the Netherlands and Germany.^22,32,33,34^ The rising number of cases was attributed to the increase in the number of travelers toward endemic areas or to population migrations for economic or political reasons (refugees). The balance between these different mechanisms may explain the complexity of the temporal patterns observed in several countries with imported malaria.

The increasing proportion of African VFRs in our study supports previous trends observed in the United States, where African VFRs increased from 17% in 1995 to 70.3% in 2015.^33^ Our finding adds to these data, since African VFRs, or more globally African individuals, are more exposed to malaria most often owing to longer stays in high-risk areas.^9,12,13,35^ Moreover, 3332 of 4199 of imported malaria cases (79.4%) reported in France from 2014 through 2016 were attributed to African VFRs compared with 777 imported malaria cases (18.5%) for European tourists (Figure 4A). The travel conditions of these 2 groups differ in age, sex, and length of stay. European individuals are often male business travelers or tourists (82.2%), while African individuals more often travel as a family, with children of all age groups (sex ratio close to 1) and significantly longer lengths of stay (6 [IQR, 4-8] vs 4 [IQR, 2-8] weeks; *P* < .001) (Figure 3C; eTable 1 in the Supplement). These findings may have implications for the use of chemoprophylaxis to prevent malaria. Therefore, in the absence of financial support to purchase malaria chemoprophylaxis, it would be difficult to obtain drugs for those traveling with their families for longer stays, such as African VFRs.^9,14,36,37^ More complex cultural factors were mentioned to explain the lesser use of chemoprophylaxis by VFRs compared with other types of travelers.^12^ These factors could explain the lower alleged chemoprophylaxis use among African individuals.

The aging of patients with malaria from 1996 to 2016 also represents a change that deserves explanation. Studies by the French National Institute of Statistics and Economic Studies reported a 5-year increase in the median age over the study period, which only partly explains our finding.^38,39^ Further investigations are then needed to clarify this point. Overall, the difference in median age between African and European individuals in our study was mainly due to the significant differences in the age structure of the 2 populations, with more children in the African individuals category. Compared with other industrialized countries, the overall median age of 33 (IQR, 21-45) years observed in our study was close to that found in the United Kingdom (35 [IQR, 24-46] years), Spain (35.6 [IQR, 27.9-44.0] years), or Canada (33.5 [range, 1-82] years).^33,40,41^

The significant increase in severe malaria incidence in our findings was also reported in other countries in Europe, as well as in Canada and the United States. This elevation could be related to the increase in age of travelers, as observed in the United Kingdom and United States.^34,40,42^ The reduction of malaria immunity in Africans living in France may have played a role; however, there is a lack of data about this variable.^43^ Considering the higher incidence of severe cases, the concomitant stability in the number and proportion of deaths is surprising. The improvement of medical care in intensive care units in the past 20 years and the use of parenteral artesunate as the first-line treatment for severe malaria since May 2011 may have contributed to the stability of the mortality.^44^ In addition, changes in the population structure of patients with imported malaria could be a further explanation, since the risk of death has been shown to be lower in African than European individuals with severe malaria.^43,45,46^ Moreover, if there is evidence for acquiring partial immune protection when an individual is subjected to repeated infections with *Plasmodium* for several years,^47,48,49^ due to several genetic specificities (glucose-6-phosphate dehydrogenase–deficient erythrocytes, type O blood group; hemoglobin AS, hemoglobin CC, and hemoglobin AC; and homozygous and heterozygous α-thalassemia), African individuals seem to be better protected against severe malaria than other groups.^50,51,52^

### Limitations

To our knowledge, our database is the largest regarding imported malaria in industrialized countries; however, the study may have limitations. First, missing data on purpose of travel and country of residence led to the exclusion of 9289 malaria cases. Therefore, a sensitivity analysis showed that the magnitude of differences when comparing results with or without excluded cases were not clinically relevant (eTable 2 in the Supplement). Second, the sensibility of our surveillance system ranged from 50% to 55% of all malaria cases diagnosed in metropolitan France—half of the imported malaria burden.^1,2,3^ However, the stability and the representativeness of our network over the study period support our findings in the changing in epidemiologic characteristics of in France. Third, because we are analyzing only confirmed malaria cases, our results cannot be extrapolated to all travelers to malaria endemic areas (eg, proportion of malaria among patients alleging prophylaxis intake).

## Conclusions

Because age appears to be associated with an increase in the severity and mortality of malaria, especially in older male European travelers, attention measures to prevent and quickly detect imported malaria in travelers should be strengthened. Moreover, the substantial increase in the proportion of African VFRs underscores the importance for public authorities to implement effective solutions and for travel operators and family practitioners to renew efforts to engage this group in the use of malaria preventive means, such as prevention messages, personal antivector protection and chemoprophylaxis. To address these issues, future surveys should be carried out to clarify travelers’ attitudes toward key malaria prophylaxis measures, to precisely identify barriers to their use, and to determine better ways to overcome these obstacles.

## Notes

Supplement. **eMethods.** Estimation of the Total Number of Malaria Cases **eFigure 1.** Boxplot Analysis Showing Monthly Distribution of Imported Malaria Cases in Civilian Travelers by Species in Metropolitan France, 1996-2016 **eFigure 2.** Ten Endemic Regions Most Visited by Imported Malaria Cases Patients, Metropolitan France, 1996-2016 **eFigure 3.** Imported Malaria Cases by Class Age by Years (N = 42,971), Metropolitan France, 1996-2016 **eFigure 4.** Median Time Between Onset Of Symptoms and Diagnosis With the 25th and 75th Percentiles for Plasmodium Species by Year, 1996-2016 **eTable 1.** Distribution of the Length of Stay for African and European Individuals Diagnosed With Imported Malaria, Metropolitan France, 1996-2016 **eTable 2.** Sensitivity Analysis Comparing the Characteristic Trends of the 9,289 Excluded Cases to the 43,333 Remained Cases, Metropolitan France, 1996-2016
