# Supplementary material for: Epidemiologic Trends in Malaria Incidence Among Travelers Returning to Metropolitan France, 1996-2016
Source: JAMA Netw Open. 2019 Apr 5;2(4):e191691. doi: 10.1001/jamanetworkopen.2019.1691 (PMC6523451; doi:10.1001/jamanetworkopen.2019.1691)
Supplement: Supplement. — eMethods. Estimation of the Total Number of Malaria Cases eFigure 1. Boxplot Analysis Showing Monthly Distribution of Imported Malaria Cases in Civilian Travelers by Species in Metropolitan France, 1996-2016 eFigure 2. Ten Endemic Regions Most Visited by Imported Malaria Cases Patients, Metropolitan France, 1996-2016 eFigure 3. Imported Malaria Cases by Class Age by Years (N = 42,971), Metropolitan France, 1996-2016 eFigure 4. Median Time Between Onset Of Symptoms and Diagnosis With the 25th and 75th Percentiles for Plasmodium Species by Year, 1996-2016 eTable 1. Distribution of the Length of Stay for African and European Individuals Diagnosed With Imported Malaria, Metropolitan France, 1996-2016 eTable 2. Sensitivity Analysis Comparing the Characteristic Trends of the 9,289 Excluded Cases to the 43,333 Remained Cases, Metropolitan France, 1996-2016 [file jamanetwopen-2-e191691-s001.pdf]

Supplementary Online Content

Kendjo E, Houzé S, Mouri O, et al; French Imported Malaria Study Group. Epidemiologic trends in malaria incidence among travelers returning to metropolitan France, 1996-2016. *JAMA Netw Open*. 2019;2(4):e191691. doi:10.1001/jamanetworkopen.2019.1691

eMethods. Estimation of the Total Number of Malaria Cases

eFigure 1. Boxplot Analysis Showing Monthly Distribution of Imported Malaria Cases in Civilian Travelers by Species in Metropolitan France, 1996-2016

eFigure 2. Ten Endemic Regions Most Visited by Imported Malaria Cases Patients, Metropolitan France, 1996-2016

eFigure 3. Imported Malaria Cases by Class Age by Years (N=42,971), Metropolitan France, 1996-2016

eFigure 4. Median Time Between Onset Of Symptoms and Diagnosis With the 25th and 75th Percentiles for Plasmodium Species by Year, 1996-2016

eTable 1. Distribution of the Length of Stay for African and European Individuals Diagnosed With Imported Malaria, Metropolitan France, 1996-2016

eTable 2. Sensitivity Analysis Comparing the Characteristic Trends of the 9,289 Excluded Cases to the 43,333 Remained Cases, Metropolitan France, 1996-2016

This supplementary material has been provided by the authors to give readers additional information about their work.

© 2019 Kendjo E et al. *JAMA Network Open.*

eMethods. Estimation of the Total Number of Malaria Cases

The total incidence of *Plasmodium* Malaria Infection was derived using four nationally exhaustive surveys performed on years 1997, 1999, 2004 and 2013 (see supplementary material for details)"

In the supplementary material: "Given the malaria case definition, which only considers cases validated by a detection of the parasite in the laboratory (optical microscopy or molecular biology), it was decided to carry out regularly retrospective questionnaires. These questionnaires were sent to all the medical laboratories belonging to the National Quality Control in Parasitology (a regulatory obligation for all laboratories that make the diagnosis of malaria) in collaboration with the National Agency for the Safety of Medicinal Products (ANSM). To consider the cases of medical laboratories that did not respond the questionnaire the following calculation formula has been applied

$N = N1 + N1'$

with $N1 = NC + NH$ and $N1' = NC' + NH'$

Where N = Estimate number of malaria cases for metropolitan France; N1 = the number of malaria cases transmitted by all medical laboratories in metropolitan France (private sector or public sector) that did answer the questionnaire; NC = the number of malaria cases transmitted by private sector laboratories (city medical laboratories or "C") that did answer the questionnaire; NH = the number of malaria cases transmitted by public sector laboratories (hospital medical laboratories or "H", mainly regional or teaching hospitals) that did answer the questionnaire; N1'= the estimated number of malaria cases for all medical laboratories (private sector or public hospitals) that did not answer the questionnaire; NC' = the number of malaria

© 2019 Kendjo E et al. *JAMA Network Open.*

cases estimated for private sector laboratories that did not answer the questionnaire and NH' = the number of malaria cases estimated for public sector laboratories that did not answer the questionnaire.

The estimate number of malaria cases, for a laboratory (type C or H) that did not answer the questionnaire, was the median of the number of malaria cases reported by laboratories (type C or H respectively), that did answer the questionnaire in the region of location of the considered laboratory.

These surveys allowed us to obtain a reference value that makes it possible to calculate the percentage of cases collected by our network for this year. This reference value was then used to adjust the representativity of the network in the following years and to provide an estimated value of malaria cases each year for metropolitan France. The following survey allows us to check for system drift.

© 2019 Kendjo E et al. *JAMA Network Open.*

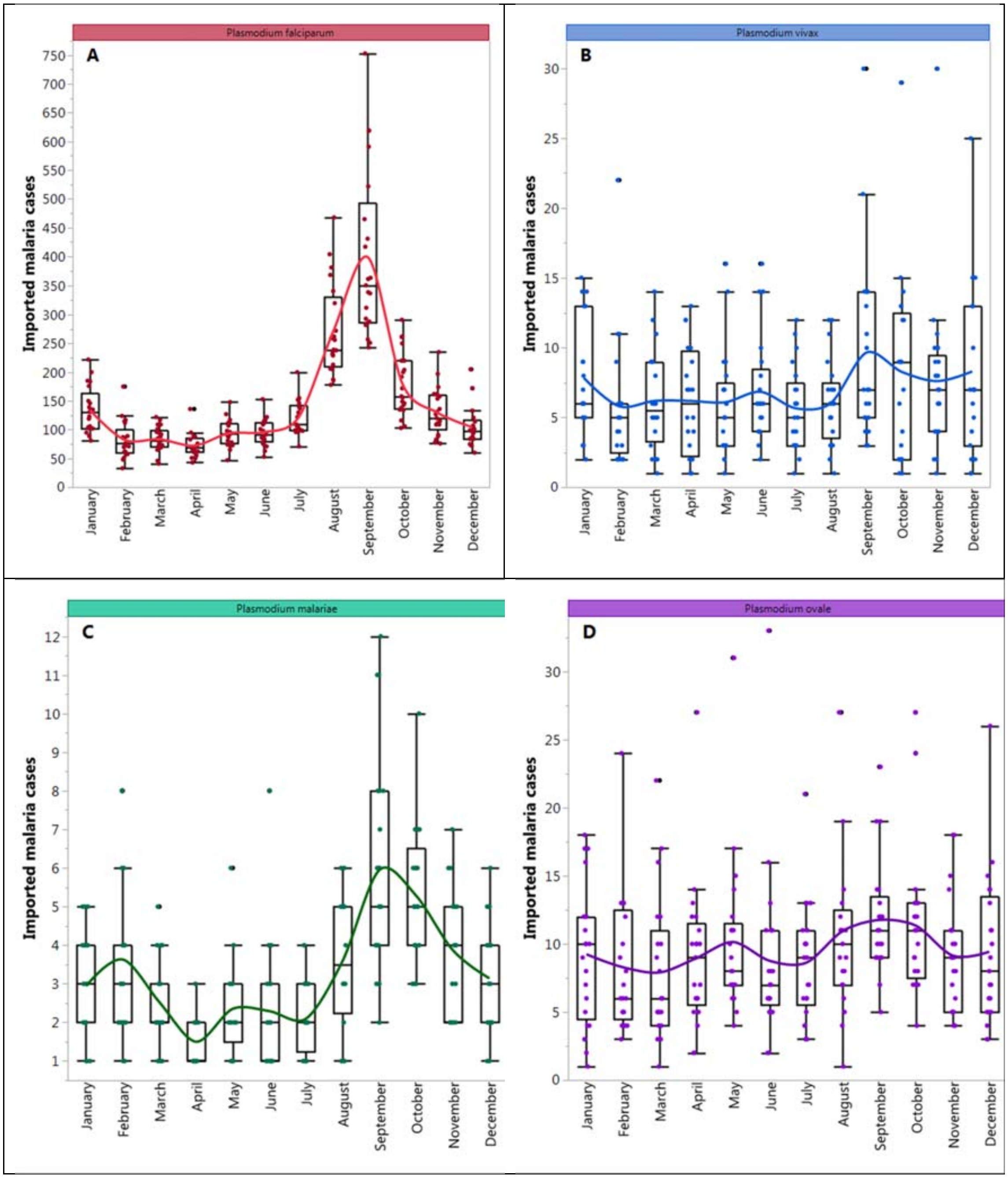

eFigure 1. Boxplot Analysis Showing Monthly Distribution of Imported Malaria Cases in Civilian Travelers by Species in Metropolitan France, 1996-2016. The line represents the median, the box, the interquartile ranges and the smooth curve using nonparametric density estimation (kernel density estimation). Mixed infections were dropped from the figure.

© 2019 Kendjo E et al. *JAMA Network Open.*

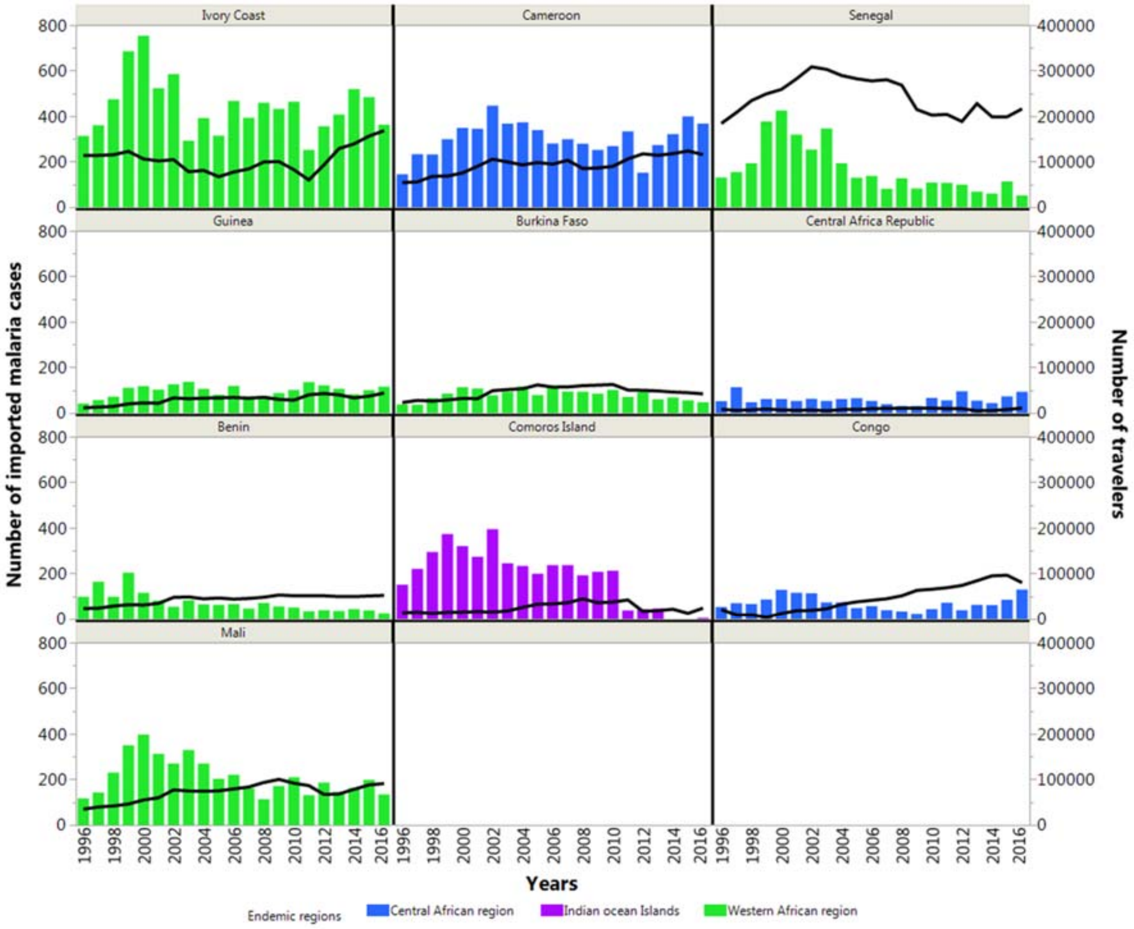

eFigure 2. Ten Endemic Regions Most Visited by Imported Malaria Cases Patients, Metropolitan France, 1996-2016.

© 2019 Kendjo E et al. *JAMA Network Open.*

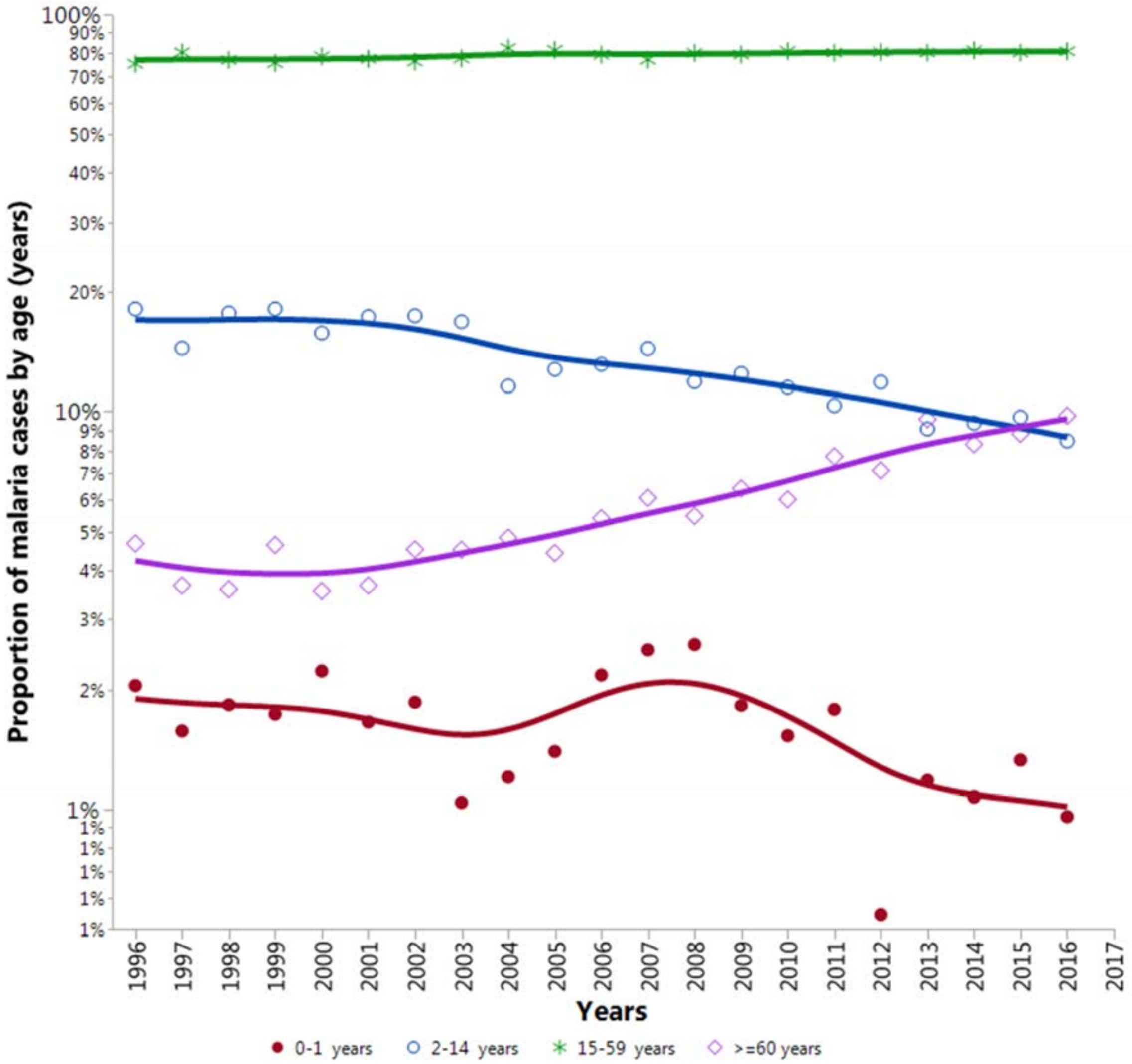

eFigure 3. Imported Malaria Cases by Class Age by Years (N=42,971), Metropolitan France, 1996-2016.

© 2019 Kendjo E et al. *JAMA Network Open.*

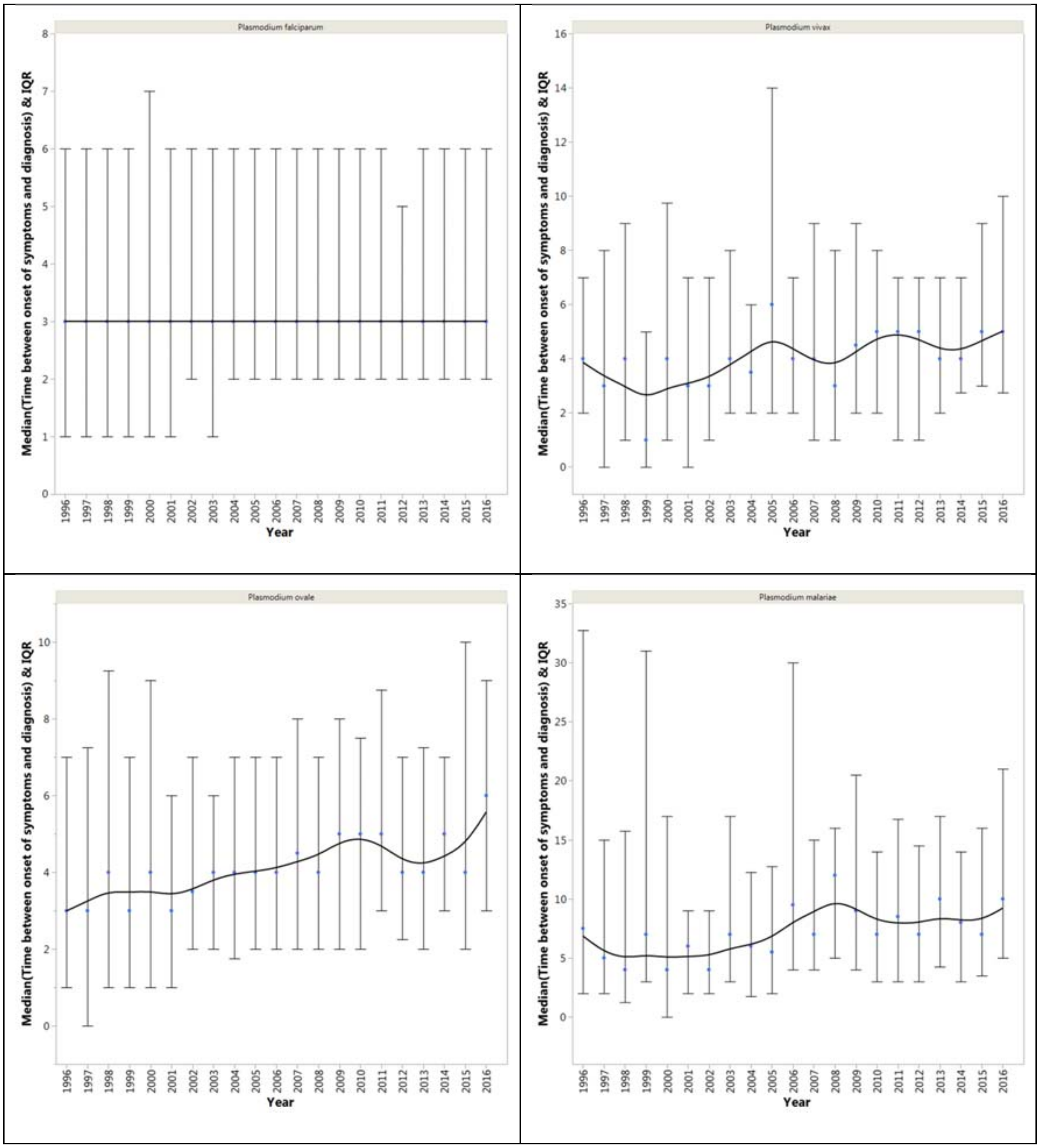

eFigure 4. Median Time Between Onset Of Symptoms and Diagnosis With the 25th and 75th Percentiles for *Plasmodium Species* by Year, 1996-2016.

© 2019 Kendjo E et al. *JAMA Network Open.*

eTable 1. Distribution of the Length of Stay for African and European Individuals Diagnosed With Imported Malaria, Metropolitan France, 1996-2016.

	**European tourists**	**African VFRs**	**Euopean individuals**	**African individuals**
N	3,033	24,312	5591	25897
**Median**	**4**	**6**	**4**	**6**
Quantiles25	2	4	2	4
Quantiles75	6	8	8	8

Length of stay was significantly longer for African VFRs (6 [4-8] weeks) compared to European tourists (4 [2-6] weeks) (p=0.03 after Bonferroni correction)

Length of stay was significantly longer for African (6 [4-8] weeks) compared to European individuals (4 [2-8] weeks) (p<0.0001).

© 2019 Kendjo E et al. *JAMA Network Open.*

eTable 2. Sensitivity Analysis Comparing the Characteristic Trends of the 9,289 Excluded Cases to the 43,333 Remained Cases, Metropolitan France, 1996-2016.

	Without missing n=43,333		With missing n=52,622	
**Sex**	**N**	**%**	**N**	**%**
Female	16,268	37.6%	19,032	36.2%
Male	26,994	62.4%	33,502	63.8%
All	43,262	100.0%	52,534	100.0%
**Ethnicity**				
African individuals	28,658	71.5%	32,632	68.1%
European individuals	10,618	26.5%	13,993	29.2%
Other individuals	783	2.0%	1,310	2.7%
All	40,059	100.0%	47,935	100.0%
**Plasmodium falciparum**				
No	5,056	11.8%	7,170	13.8%
Yes	37,065	88.2%	44,969	86.2%
All	42,850	100.0%	52,139	100.0%
**Plasmodium vivax**				
No	40,962	95.6%	49,311	94.6%
Yes	1,732	4.4%	2,828	5.4%
All	42,850	100.0%	52,139	100.0%
**Plasmodium ovale**				
No	40,108	93.6%	48,460	92.9%
Yes	2,405	6.4%	3,679	7.1%
All	42,850	100.0%	52,139	100.0%
Mixed infection				
No	42,114	98.3%	51,305	98.4%
Yes	736	1.7%	834	1.6%
All	42,850	100.0	52,139	100.0%
**Plasmodium spp**				
No	42,737	99.7%	51,991	99.7%
Yes	113	0.3%	148	0.3%
All	42,850	100.0%	52,139	100.0%
**Alleged chemoprophylaxis**				
No	22,404	55.8%	27,050	56.3%
Yes	17,712	44.2%	20,969	43.7%
All	40,116	100.0%	48,019	100.0%
**Severity**				
Severe malaria	5,158	11.9%	6,006	11.4%
Uncomplicated malaria	38,175	88.1%	46,615	88.6%
All	43,333	100.0%	52,621	100.0%
**Clinical outcome**				
Alive	43,167	99.6%	52,428	99.6%
Dead	166	0.4%	194	0.4%
**Age (years)**, median [25th percentile; 75th percentile]	33	[21; 45]	32	[21; 44]

© 2019 Kendjo E et al. *JAMA Network Open.*
